# Can hydrophilic coated catheters be beneficial for the public healthcare system in Brazil? - A cost-effectiveness analysis in patients with spinal cord injuries

**DOI:** 10.1590/S1677-5538.IBJU.2017.0221

**Published:** 2018

**Authors:** José Carlos Truzzi, Vanessa Teich, Camila Pepe

**Affiliations:** 1 Escola Paulista de Medicina, Universidade Federal de São Paulo, SP, Brasil; 2 Hospital Alemão Oswaldo Cruz, São Paulo, SP, Brasil; 3 Sense Company, São Paulo, SP, Brasil

**Keywords:** Cost-Benefit Analysis, Spinal Cord Injuries, Intermittent Urethral Catheterization, Urinary Tract Infections

## Abstract

**Introduction:**

Detrusor sphincter dyssynergia affects 70% to 80% of all spinal cord injury patients, resulting in increased risk of urinary tract infections (UTIs) and potential exposure to antimicrobial resistance. In Brazil, local guidelines recommend intermittent catheterization as the best method for bladder emptying, and two catheter types are available: the conventional uncoated PVC and the hydrophilic coated catheters.

**Objective:**

To evaluate the cost-effectiveness of two types of catheters for intermittent catheterization from the perspective of the Brazilian public healthcare system.

**Materials and Methods:**

A Markov model was used to evaluate cost-effectiveness in those with spinal cord injuries. A primary analysis was conducted on all possible adverse events, and a secondary analysis was performed with urinary tract infections as the only relevant parameter. The results were presented as cost per life years gained (LYG), per quality-adjusted life years (QALY) and per number of urinary tract infections (UTIs) avoided.

**Results:**

The base scenario of all adverse events shows a cost-effective result of hydrophilic coated catheters compared to uncoated PVC catheters at 57,432 BRL (Brazilian Reais) per LYG and 122,330 BRL per QALY. The secondary scenario showed that the use of hydrophilic coated catheters reduces the total number of UTIs, indicating that an additional cost of hydrophilic coated catheters of 31,240 BRL over a lifetime will reduce lifetime UTIs by 6%.

**Conclusions:**

Despite the higher unit value, the use of hydrophilic coated catheters is a cost-effective treatment from the perspective of the Brazilian public healthcare system.

## INTRODUCTION

Every single year, almost 10,000 Brazilians suffer from a spinal cord injury (SCI) — an injury that comes with great cost for the individual as well as for the public healthcare system (1, 2). As a consequence of the injury, 70% to 80% are diagnosed with detrusor sphincter dyssynergia, a dysfunction that leads to incomplete bladder emptying (3). National and International guidelines recommend bladder emptying with intermittent catheterization (IC) 4-6 times per day (4-6). Among the benefits of IC are the preservation of the structure and function of the upper urinary tract, reduced risk of vesicoureteral reflux, improvement of urinary continence and reduction of urinary tract infections (UTI). These benefits allow SCI patients to be more independent and have a better quality of life (4). Paradoxically, the most prominent consequence of IC is the recurrence of UTIs. In Brazil, quinolones are the first line of antibiotics used to treat UTIs. This treatment involves a resistance level above 30% in some regions (7).

In Brazil, conventional uncoated polyvinylchloride (PVC) catheters, which require manual application of lubricant to ease and reduce friction, are the most frequently used catheters. Alternative catheters available in Brazil are the pre-lubricated hydrophilic coated ones. These catheters have a surface coating, which binds with water to form an even coating, thereby eliminating the need for manual application. At the same time, it most likely poses a lower risk of UTIs and hematuria to SCI patients.

The aim of this study is to evaluate the cost-effectiveness of these two different types of catheter for IC in a lifetime perspective. The structure is based on an international published model (8) with adaptations to Brazilian costs and the healthcare system.

## MATERIALS AND METHODS

### Structure of the Model

**Figure 1:**
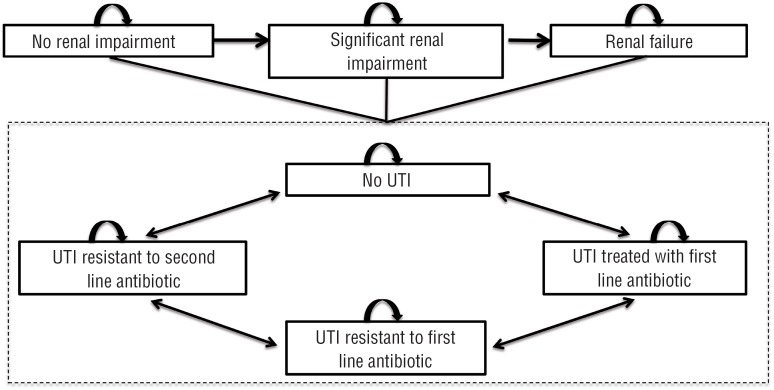
Markov decision model - three renal stages and four urinary tract infection (UTI) levels regardless the impairment of renal function.

A Markov decision model was adjusted to Brazilian reality to evaluate the cost-effectiveness of hydrophilic coated versus uncoated PVC catheters used by SCI patients in Brazil. The analysis was performed using the Excel^®^ 2013 software to simulate, in monthly cycles, IC-related complications from the first catheterization until death (lifetime) (Figure-1). For the primary analysis, all adverse events were taken into consideration, whereas the secondary analysis covered UTI as the single adverse event.

Based on the original study by Clark et al., SCI patients suffer from chronic urinary retention (80% males, 20% females, average age 36 years old) (8).

A consequence of urinary retention over a long period of time is the impairment of renal function. To reflect this reality, three renal stages were established in relation to its impact on the renal function: “No impairment”, “Significant impairment” and “Renal failure”. It is assumed that all new IC users will have “No impairment” and that it is not possible to recover or regress to a previous stage of renal function, but only progression of stages (Figure-1).

UTI is the most common complication of IC, and the current model consists of four different levels of UTIs that the SCI patient can go through. This is a modification compared to the original model by Clark et al., however it was considered relevant to describe the treatment pathways in Brazil. (8) In this study, we defined the following UTI levels: “No UTI”, “UTI treated with first line antibiotics”, “UTI resistant to first line antibiotics” and “UTIs resistant to second line antibiotics”. All new IC users are simulated to have “No UTI”. Regardless of impairment of renal function, SCI patient remains subject to the same UTI risk and treatment (Figure-1).

Additional adverse events considered as consequences of IC were the development of sepsis, urethral injury during catheterization and subsequent urethral stricture, and formation of stones within the urinary bladder.

### Data input

The development of UTIs was considered the key parameter in the model. A systematic review of the literature was carried out with keywords and MeSH that covered Spinal Cord Injuries, Hydrophilic Coated Catheters, Economics, Costs, Cost Analysis, and related terms for both English and Spanish languages in the following databases: The Cochrance Library, MEDLINE via Pubmed, Literatura Latino-americana e do Caribe em Ciências da Saúde (LILACS), and Centre for Reviews and Dissemination (CRD). Further research was conducted on the websites of health technology assessment agencies and related institutions and their databases. Digital searches were complemented by manual searches of bibliographic references and abstracts of selected publications.

This search resulted in identifying seventy-seven studies. Of these, only randomized clinical trials, meta-analysis, reviews, observational studies and economic evaluations published before May 2016 were selected. Eighteen studies met these inclusion criteria and were submitted for a complete review. The PICO (Population/Intervention/Comparison/Outcome) criteria were used for the structured question. Finally, four studies evaluating hydrophilic coated and uncoated PVC catheters met the criteria and were used for the cost-effectiveness study. The baseline risk of UTI was based on data from De Ridder et al. (2005) (9), Cardenas et al. (2009) (10), Cardenas et al. (2011) (11), and Sarica et al. (2010) (12). Individual data from each study were compiled to calculate the UTI incidence of both catheters. The relative risk (RR) of UTI per month between hydrophilic and PVC catheters was calculated for each one of the four studies mentioned above. A weighted average of the relative risks was calculated and based on it and the reduction risk of UTI was determined. (Table-1).

**Table 1:** Urinary tract infection responding to initial antibiotic treatment.

Source	Catheter type	Patients (N)	UTI Incidence	Mean Followup months	UTIs per month	Weighted average 1	Relative Risk (RR): Hydrophilic coated vs PVC
**UTI rates in the uncoated PVC catheter population**							
Cardenas et al. (2011) ^a^	Conventional PVC	114	-	3-6	0.48	54.49	-
Sarica et al. (2010) ^b^	Conventional PVC	21	4.00	1.5	2.67	56.00	-
Cardenas et al. (2009) ^c^	Conventional PVC	23	1.65	12	0.14	3.16	-
De Ridder et al. (2005) ^d^	Conventional PVC	61	-	12	0.38	23.18	-
	**Total**	**219**					
**UTI rates in the hydrophilic coated catheter population**							
Cardenas et al. (2011) ^a^	Hydrophilic coated	105	-	3-6	0.48	50.30	1.00
Sarica et al. (2010) ^b^	Hydrophilic coated	21	1.00	1.5	0.67	14.00	0.25
Cardenas et al. (2009) ^c^	Hydrophilic coated	22	0.77	12	0.06	1.41	0.47
De Ridder et al. (2005) ^d^	Hydrophilic coated	61	-	12	0.34	20.74	0.89
	**Total**	**209**					
**Hydrophilic-coated versus conventional uncoated pvc catheter treatment effect**							**0.84 (16% reduction)** 2

1 number of patients x UTI per month

2 mean relative risk weighted by the number of patients in each study - (RR a X N a) + (RR b X N b) + (RR c X N c) + (RR d X N d) / (N a + N b + N c + N d)

The analysis was limited to bladder voiding through intermittent catheterization in the SCI population. Other methods for voiding dysfunction management, such as indwelling catheters or urostomies were not included, as they are associated with different risk profiles. We focused on IC, once the Brazilian Society of Urology recommends this treatment for long-term urinary retention patients with neurogenic voiding dysfunction.

For bacterial resistance, the first line treatment was based on ciprofloxacin. The resistance rate of 34% was determined by D'Addazio et al. (7). As second-line antibiotic, acetyl cefuroxime was adopted as the drug of choice for oral use in cases of failure in the first-line of antimicrobial treatment with a resistance level of 3.4% (13). A third-line antibiotic treatment was defined as ceftriaxone, through parenteral administration. The studies evaluated antibacterial resistance to community-acquired UTIs. For bacterial resistance rate to antibiotics, it was decided to use data outside of hospital settings only.

All other complications included in the model can be found in Table-2. For these complications, the keywords and MeSH used in the literature search were the same previously described for the same database. Due to a limited number of studies regarding these other complications and their relation to different types of catheter, we decided to include observational studies, which resulted in three additional publications as listed on Table-2 (14-16).

**Table 2:** Key input parameters.

Parameters		Value	Source/assumption
**Monthly adverse event rates (conventional PVC)**			
	Urinary tract infections	62.48%	De Rider et al. (2005), Cardenas et al. (2009) Cardenas et al. (2011), Sarica et al. (2010)
	Bladder stones	0.12%	Perrouin-Verbe et al. (1995), Chai et al. (1995)
	Kidney stones	0.12%	Assumed same bladder stones
	Urethral injury	0.19%	Perrouin-Verbe et al. (1995), Chai et al. (1995)
	Urosepsis	0.32%	Perrouin-Verbe et al. (1995), Chai et al. (1995), Weld et al. (2000)
**Treatment effect (hydrophilic coated vs PvC)**			
	Urinary tract infections	0.84	Table 1
	Bladder stones	0.90	Clark et al. (2016)
	Kidney stones	0.90	Clark et al. (2016)
	Urethral injury	0.90	Clark et al. (2016)
	Urosepsis	0.90	Clark et al. (2016)
**Costs**			
	Monthly cost, PVC catheter*	R$ 74.27	Banco de Preços em Saúde (19)
	Monthly cost, hydrophilic coated catheter*	R$ 608.27	Market Price
	Monthly cost, lubricant*	R$ 132.75	Banco de Preços em Saúde (19)
	Urinary tract infections, per event	R$ 554.16	SIGTAP/ TABNET (20, 21)
	Urinary tract infections, antibiotics**	R$ 60.50	Banco de Preços em Saúde (19)
	Urosepsis	R$ 708.36	SIGTAP/ TABNET (20, 21)
	Urethral injury	R$ 605.33	SIGTAP/ TABNET (20, 21)
	Kidney stones	R$ 524.30	SIGTAP/ TABNET (20, 21)
	Bladder stones	R$ 721.95	SIGTAP/ TABNET (20, 21)
	Major renal impairment, per month	R$ 82.60	SIGTAP/ TABNET (20, 21)
	Renal failure, per month	R$ 2,589.02	SIGTAP/ TABNET (20, 21)
**Utility decrements**			
	Urinary tract infection	0.060	NICE Model (17)
	Urinary tract infection, resistant	0.104	NICE Model (17)
	Kidney stones	0.050	Clark et al. (2015)
	Bladder stones	0.050	Clark et al. (2015)
	Urethral injury	0.104	NICE Model (17)
	Urosepsis	0.160	NICE Model (17)
	Major renal impairment	0.155	NICE Model (17)
	Renal failure	0.250	NICE Model (17)
**Mortality multipliers**			
	Urinary tract infection	0.000	Model structure
	Urinary tract infection, resistant***	145.270	Clark et al. (2015)
	Urinary tract infection, weighted****	49.390	Calculated based on Schito et al. (2009) (13)
	Urosepsis	797.600	Clark et al. (2015)
	Major renal impairment	18.000	Clark et al. (2015)
	Renal failure	54.000	Clark et al. (2015)

* Assuming the practice of four catheterizations per day for bladder emptying and the use of half a tube of lubricant per catheterization (2 tubes per day)

** Antibiotic therapy – 1^st^ line (ciprofloxacin 500 mg every 12h for 7 days, oral use; resistance rate = 34%); Antibiotic therapy – 2^nd^ line (cefuroxime 500 mg every 12h for 7 days, oral use; resistance rate = 3.4%) and Antibiotic therapy – 3^rd^ line (ceftriaxone 1 g every 12h for 7 days, parenteral).

*** Resistance rate to ciprofloxacin = 34%.

**** s 145.27*34%* + 0.00*66%

Within the adopted model, a state of quality of life that follows each adverse event (utility) was included. We decided to use the original data from The National Institute of Health and Care Excellence (NICE), since there is no data available for the Brazilian population (17). For mortality rates, the 2014 Brazilian mortality board of Instituto Brasileiro de Geografia e Estatística (IBGE) (18) was used. The adjustment factors were based on Clark et al. (8) and D'Addazio et al. (7).

### Cost Data

Costs related to UTI treatments, as well as the necessary materials for catheterization, were obtained from the Banco Nacional de Preços (19), except for the hydrophilic coated catheters, which were provided by a company in Brazil, considering the lowest individual price among catheters registered and approved by ANVISA (Brazilian Health Regulatory Agency). The consultation of a single local source (Banco Nacional de Preços) regarding the costs related to urinary tract infection was determined by the fact of being the aim of the current study to evaluate the cost-effectiveness of hydrophilic catheters from the perspective of the public healthcare system in Brazil. Banco Nacional de Preços is the sole official source used by the Brazilian government for budget calculation. The use of foreign countries’ cost data could interfere with the analysis and results presented here.

Costs due to lubrication, required when using uncoated PVC catheters, were considered as reusable lubricant, since this is the most common practice in Brazil, whilst the unit value of each lubricant package was divided by the average volume used in each procedure, for a mean of four catheterizations per day. As indicated by the manufacturer, the volume to be inserted into the urethra before each catheterization is estimated at 15 grams for men and 3 to 5 grams for women. In order to apply the relative proportion between sexes in the present study, it was estimated the use of two tubes a day for a total of four catheterizations.

The costs related to procedures, diagnostic exams and treatment of adverse events were obtained from the SIGTAP table (20). The hospitalization costs were obtained from TABNET data (21). Both SIGTAP and TABNET correspond to the official sites for consultation adopted by the Brazilian government in the public healthcare system. For calculation purposes, patients who reach the hospitalization stage cost the public healthcare system approximately 525.60 BRL (Brazilian Reais) per UTI, and progression to sepsis costs 708.36 BRL. The list of all costs and sources are presented in Table-2.

### Output

Direct medical costs, life years gained (LYG), quality adjusted life years (QALY) and the number of UTIs avoided were calculated from the perspective of the Brazilian public healthcare system. An annual discount of 5% was applied to costs, QALY and LYG, but UTIs were not discounted. All results are reported as incremental cost-effectiveness ratio (ICER). A deterministic one-way sensitivity analysis of parameters was performed to assess the robustness of the variables in the model. Three parameters were considered key to results and deterministic analysis: amount of lubricant, resistance rates to antibiotics and UTI rates.

No official threshold for ICER exists for the Brazilian population. In the UK, this figure is in the range of £20,000—£30,000 per QALY, and in the United States the threshold is US$50,000. In Brazil, the ICER threshold was estimated by Prado as 147,000 BRL and this was the ICER threshold adopted for the evaluation of results in this cost-effectiveness study (22).

## RESULTS

Based on the studies from De Ridder et al. (2005) (9), Cardenas et al. (2009) (10), Cardenas et al. (2011) (11), and Sarica et al. (2010) (12), the monthly risk of developing UTI while using uncoated PVC catheters was estimated in 62%, with a reduction by 16% while using hydrophilic coated catheters (Table-1).

Within a base case primary analysis, the model predicts that with an additional cost of 31,221 BRL per SCI patient within a life perspective, an IC user can live an additional 0.54 years with the use of hydrophilic coated catheters. In a Brazilian setting, the hydrophilic coated catheter is considered cost-effective at a level of 122,330 BRL per QALY for the base scenario (Table-3).

**Table 3:** Cost-effectiveness results of primary analysis (all adverse events).

	Cost (BRL)	QALYs	LYG	UTI
Conventional PVC	17,255	2.550	5.689	54.73
Hydrophilic coated	48,476	2.805	6.233	51.53
Incremental values	31,221	0.255	0.544	-3.20
ICER (BRL/QALY gained)		122,330 BRL per QALY		
ICER (BRL/LYG)		57,432 BRL per LYG		

For the secondary analysis, considering only UTI as an adverse event related to IC, the ICER values of 57,468 BRL per LYG and 122,406 BRL per QALY were also considered cost effective in the Brazilian setting (Table-4). For the secondary analysis with UTI as the only parameter, the results indicate the need for an investment of approximately 9,778 BRL per avoided UTI. Additionally, the user will have a 6% reduction of UTIs requiring treatment and an increase in QALY by 0.25 within a life perspective.

**Table 4:** Cost effectiveness results of secondary analysis (UTIs).

	Cost (BRL)	QALYs	LYG	UTI
Conventional PVC	17,255	2.550	5.689	54.73
Hydrophilic coated	48,495	2.805	6.233	51.53
Incremental values	31,240	0.255	0.544	-3.20
ICER (BRL/QALY gained)		122,406 BRL per QALY		
ICER (BRL/LYG)		57,468 BRL per LYG		
ICER (BRL/UTI avoided)		9,778 BRL saved per UTI avoided		

Three parameters were identified relevant to investigate in a deterministic one-way sensitivity analysis (deterministic analysis): the lubrication, the bacteria resistance level and the UTI rate. ANVISA recommends that lubricants for uncoated catheters are single-use (23). Therefore, a one-way sensitivity analysis increases the number to four lubricants per day. By implementing the ANVISA recommendation, the incremental cost was reduced significantly to R$ 86,831 per QALY. Implementing a conservative resistance rate for ciprofloxacin of 16.5% (24), results were still considered cost-effective at R$ 122,527 per QALY. For UTI varying rates, it was decided to include results from a literature review carried out by Li et al. (25), and one scenario based on the study that has been conducted over a period of 10+ years on IC users by Cardenas et al. (10). Adopting different UTI rates, the levels implemented in the base scenario are highly cost-effective from 40,188 BRL to 76,796 BRL (Table-5).

**Table 5:** Deterministic univariate sensitivity analysis.

Parameters	Value tested	ICER (BRL/QALY)	Source
Deterministic results		122,330	-
**Lubricant tube per day**			
Base case	2	-	Table 2
Alternative	4	86,831	ANVISA guideline
	1	140,079	Assumed
**Resistance level for antibiotics**			
Base case	34%	-	D'Addazio et al. (2015), Table 2
Alternative	16%	122,527	Kiffer et al. (2011)
	45%	122,206	Assumption
**UTI reduction** *			
Base case	16%	-	Table 1
	26%	76,796	Li et al. (2013)
Alternative	53%	40,188	Community data, Cardenas et al. (2009)

* The studies come with different UTI rates based on published information

## DISCUSSION

The most frequent complication of intermittent catheterization is urinary tract infection. A 12-year follow-up study showed that chronic or recurrent UTIs are as frequent as 42% in patients with IC (26). Throughout the last two decades, the increasing rates of bacterial resistance to antibiotics have worried the scientific community, and efforts have been made to reduce UTI rates. De Ridder et al. prospectively evaluated men with neurogenic bladder dysfunction due to spinal cord injury, and observed two times fewer symptomatic infections among those using hydrophilic catheters (9). Cardenas et al. demonstrated that patients with spinal cord injury using hydrophilic catheters required less antibiotic treatment for UTIs when compared to those using PVC catheters (10). In a randomized study, also with spinal cord injury patients monitored from the beginning of the rehabilitation phase, a 21% reduction rate was observed in the risk of UTI development in the initial rehabilitation phase and a delay in the occurrence of the first symptomatic UTI event (11). Forty percent of spinal cord injury patients with intermittent catheterization with hydrophilic catheters monitored over five to nine years still passed sterile urine (27). In a systematic review with five studies involving 462 patients, the incidence of symptomatic UTI and hematuria were significantly lower in the hydrophilic catheter group than in the non-hydrophilic catheter group (25).

This study was designed to determine the cost-effectiveness of two different catheters used for patients with spinal cord injury in an intermittent catheterization program. It can provide local IC decision-makers with a tool that captures the relevant costs, consequences and benefits of two different types of catheters in a lifetime perspective. The model structure was based on a European model published in 2016, specially adapted to the Brazilian cost and public healthcare system (8). Despite no official cost-effectiveness threshold in Brazil, Prado et al. demonstrated that a threshold of up to 147,000 BRL is sufficient to consider cost-effectiveness in Brazil (22).

Considering the above-mentioned threshold, after analyzing all possible adverse events of IC (primary analysis), as well as when UTI was the sole parameter considered (secondary analysis), the use of a hydrophilic coated catheter resulted in a highly cost-effective result. The deterministic sensitivity analysis revealed that despite the amount of lubricant used, the bacteria resistance rate to antibiotics and different UTI reduction rates, the cost-effectiveness favored hydrophilic coated catheters for the Brazilian scenario.

The results match those from the original model from Europe (8), as well as a later Japanese version (28), where hydrophilic coated catheters were proved to be cost-effective despite their higher unit price. A key difference to the original model by Clark et al. (8) was the implementation of different treatment pathways in case of bacterial resistance, as well as a higher resistance level particularly to ciprofloxacin, the most commonly used antibiotic for treating UTIs in Brazil. It is well-known that the values of bacteria assumed in the current study were those based on community general population, and not specifically in patients with neurogenic bladder dysfunction. This is relevant, considering that a recent study conducted in the United States documented that SCI patients have higher rates of antibacterial resistance than those observed in the general population (29). The adopted model did not consider the important aspect of higher antibacterial resistance levels in relation to hospitals or rehabilitation centers.

It is worth noting that the inputs were conservative, as the model assumes that all SCI patients enter the model without any complications except the need for IC. SCI patients are usually very complex due to several comorbidities. However, to ensure the model is as objective as possible and for the purpose of simplification, it was decided to conduct the analysis on less complex SCI patients.

Although the practice of reusing uncoated PVC catheters in Brazil is common, according to the ANVISA, as well as included on the manufacturer's label, the catheters are not intended for anything more than single use. A recent study involving Paralympic athletes, including Brazilians, showed that those with re-used catheters presented more than twice the UTI rates compared to athletes from European countries, where hydrophilic coated catheters are the standard of care (5, 30).

According to scientific evidence, the hydrophilic-coated catheters have a lower friction force, as well as lower rates of hematuria when compared to uncoated PVC catheters (31). However, these differences were not incorporated into the model. It is believed that performing at least four to six catheterizations per day with uncoated catheters is likely to raise the likelihood of urethral trauma.

It has been documented that lack of preference for a specific catheter can reduce compliance and thereby increase the occurrence of catheter-related complications. Hydrophilic-coated catheters have been associated with greater patient satisfaction in aspects such as comfort and convenience when compared to uncoated PVC catheters (30-32). These parameters were not applicable in the structure of the current model due a lack of information regarding this subjective aspect in our country.

Regarding uncoated PVC catheters, most of them contain softeners such as phthalates that may put the users at risk. Although the exposure to phthalates risk on the part of neither patients nor healthcare professionals was incorporated into this model, numerous investigations have demonstrated that with continuous exposure to phthalates, they will accumulate in the human body and therefore affect the hormones - especially in women and children (33).

## CONCLUSIONS

Despite a difference in unit cost of the two different types of catheters, the hydrophilic--coated catheters seem to be cost-effective within a lifetime perspective for SCI patients. The results have not considered patient, urologist or other healthcare professional preference and convenience. An analysis such as a multi-criteria decision analysis would give better insight into the preferences of patients and healthcare professionals.
